# The effects of sympathetic activity induced by ice water on blood flow and brachial artery flow-mediated dilatation response in healthy volunteers

**DOI:** 10.1371/journal.pone.0219814

**Published:** 2019-09-13

**Authors:** Kristian Magnus Gundersen, Christoffer Nyborg, Øyvind Heiberg Sundby, Jonny Hisdal

**Affiliations:** 1 Section of Vascular Investigations, Department of Vascular Surgery, Division of Cardiovascular and Pulmonary Diseases, Oslo University Hospital, Oslo, Norway; 2 Faculty of Medicine, Institute of Clinical Medicine, University of Oslo, Oslo, Norway; University of Illinois at Urbana-Champaign, UNITED STATES

## Abstract

**Objective:**

To investigate the association between sympathetic activity, reactive hyperemia and brachial artery flow-mediated dilation (FMD).

**Background:**

It is claimed that major surgery has an impact on endothelial function, as observed by post-operative reduced brachial artery FMD response. However, another explanation for the observed reduced FMD response post-operatively may be sympathetic stress-induced reduction in blood flow.

**Methods:**

Seventeen healthy volunteers with a median age (25^th^-75^th^ percentiles) of 23.5 (23–24.8) years were recruited. Participants’ brachial blood flow and FMD response were measured (i) during normal non-stress conditions (Normal_1_); (ii) during exposure to ice water; and (iii) afterwards, under normal non-stress conditions (Normal_2_). We continuously measured arterial blood pressure (Finometer), heart rate (ECG), skin blood flow of the index finger (laser Doppler), and brachial artery blood flow and diameter (Ultrasound Doppler). Measurements were taken at baseline; before a 5-min suprasystolic forearm occlusion; and following a 3-min post-occlusion, to measure reactive hyperemia and FMD.

**Results:**

Median (25^th^-75^th^ percentiles) FMD response after exposure to ice water was reduced compared to non-stress conditions [4.9 (2.9–8.4) % during ice water vs. 9.7 (7.6–12.2) % Normal_1_ and 9.7 (6.4–10.3) % Normal_2_, P < 0.001]. Blood flow 60 s after cuff-deflation during ice water exposure was significantly reduced to 328 (289–421) mL compared to non-stress conditions (both P < 0.05). No differences were observed between Normal_1_ [446 (359–506) mL] and Normal_2_ [455 (365–515) mL] (both P > 0.05). Heart rate significantly increased during ice water exposure [67 (59–69) beats/min)] compared to 55 (49–60) beats/min during Normal_1_ and 54 (47–60) beats/min during Normal_2_ (both P < 0.05). MAP did not change during Normal_1_ [72 (64–84)] or during Normal_2_ [71 (65–81) mm Hg] (both P > 0.05), but increased to 86 (75–98) mm Hg during ice water exposure (P < 0.05).

**Conclusions:**

Increased sympathetic activity resulted in decreased blood flow and brachial artery FMD response in healthy volunteers, independent of endothelial dysfunction. Future studies should adjust for blood flow when interpreting the FMD response.

## Introduction

Endothelial dysfunction is associated with increased cardiovascular risk, and is an initial event in the development of atherosclerosis [1, 2]. Brachial endothelium-dependent flow-mediated dilation technique (FMD) is a non-invasive clinical research method to assess vascular endothelial function [3]. Brachial artery FMD measures vasodilation following an acute increase in blood flow (reactive hyperemia), typically induced by a period of circulatory occlusion [4]. It has been suggested that major surgery–and in particular, the use of cardiopulmonary bypass surgery (CBS)–triggers a systemic inflammatory response, resulting in endothelial dysfunction [5–11].

A recent study demonstrated, however, that cardiac surgery reduces both resting blood flow and reactive hyperemia in the arm postoperatively [12]. Further, these reductions were followed by a decrease in the FMD response among patients 24 h postoperatively, without any changes in resting artery diameter [12]. Hyperemic shear stress stimulates the FMD response, suggesting that sympathetic stress may alter endothelium-dependent vasodilation. Therefore, the reduced FMD response interpreted as an indicator of endothelial dysfunction postoperatively in previous investigations [11, 13, 14] may derive from an alternative, confounding source. Specifically, decreased hyperemic response may result from postoperative-induced sympathetic activity, rather than impaired endothelial function *per se*. Although several studies report diminished FMD response during sympathetic stimulation [15–18], the association between the magnitude of reactive hyperemia and the FMD response related to sympathetic activity has attracted little attention.

To further elucidate the effect of increased sympathetic activity on the FMD response, we measured the brachial artery FMD response in healthy young volunteers under both normal conditions and during increased sympathetic activity induced by foot exposure to ice water. Our hypothesis was that independent of endothelial dysfunction, increases in sympathetic stress would result in reduced blood flow, and consequently, decreased FMD response compared to a non-stress environment.

## Materials and methods

### Participants

Seventeen healthy volunteers (10 men, 7 women) were recruited from the university community in Oslo. All participants were between 20 and 28 years of age, physically active, non-smokers, non-hypertensive (resting blood pressure <140/80 mm Hg), non-obese (body mass index <30), and had no family history of heart disease. All participants were free of any recognized overt cardiovascular, pulmonary or metabolic diseases. Participants were not taking medications with vasoactive effects. This includes contraceptives, hormone replacement therapy, anti-hypertension, lipid-lowering and/or anti-diabetes drugs. Written informed consent was obtained from each participant prior to study participation. The experimental protocol was approved by the Regional Committees for Medical and Health Research Ethics in Norway (protocol number: 2013/613/REK) and performed in accordance with the Declaration of Helsinki.

### Preparations

The experiments were performed between 8:00 a.m. and 12:00 noon, and the participants were asked to arrive in a fasted state, abstaining from caffeine and alcohol consumption 24 h before the start of study. The participants were also instructed to refrain from engaging in intense physical activity or consuming tobacco or vitamin supplements 12 h prior to testing. Before the experiments, anthropometric parameters were measured, including height, body mass, and resting blood pressure.

### Experimental design

After 20 minutes of supine resting in a climate controlled (22–25°C) quiet room, study participants were exposed to three separate sequences of hyperemic shear stress in the following order: (i) normal rested conditions; (ii) increase in sympathetic activity, and (iii) normal rested conditions. Increased sympathetic activity was induced by submerging the right foot and leg in ice water slurry (4–5°C) from 10 s before cuff deflation to 90 s afterwards. This is a sympathetic stress test, which evokes an increase in blood pressure mainly by means of peripheral vasoconstriction [19]. The brachial FMD response was measured after each sequence. Each of the three sequences were separated by a 20-min wash‐out period between sets.

### Measurements

During the experiments, blood pressure was continuously measured from the third finger of the left hand, using a photoplethysmographic volume-clamp method (Finometer; FMS Finapres Medical Systems BV, Amsterdam, The Netherlands). Acral skin blood flux was measured using Laser Doppler Flowmetry (LDF) probes (404–1 and Periflux 4000; Perimed AB, Järfälla, Sweden) attached with double-adhesive tape on the pulp of the left index finger. LDF gives a semi-quantitative measurement of changes in cutaneous tissue, expressed in arbitrary units (AU) [20]. To enable ECG-gated acquisition of the flow and flux images, heart rate was monitored continuously with a 3-lead ECG connected to custom-made software for physiological data (REGIST 3, Morten Eriksen, University of Oslo, Oslo, Norway).

Brachial artery FMD was measured in the right arm according to current expert consensus guidelines [4]. Briefly, baseline diameter and central hemodynamics were recorded 60 s before each cuff inflation. Blood flow velocity was estimated by time-averaging the Doppler signal from a mid-artery sample volume. Thereafter, ischemia was induced through rapid inflation of the pneumatic cuff to a minimum pressure of 250 ± 5 mm Hg for 5 min before cuff deflation. After a 5-min interval of ischemia, rapid cuff deflation induced a brief high-flow state (hyperemic shear stress). We recorded Doppler signals continuously for three minutes after cuff deflation to capture the maximal vasodilation response and reappearance of blood flow to baseline values. During the blood flow measurements, a Doppler beam-vessel insonation angle was kept at <60° to ensure valid velocity calculations.

**Fig 1:**
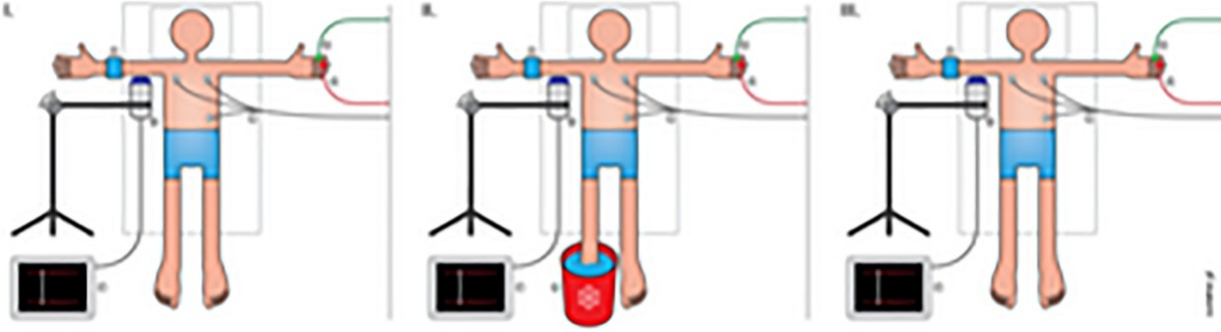
The experimental setup. I: Illustration of the experimental setup with probes attached to the upper extremites. I-II-III: A, a distal occlusion cuff attached to the lower right arm; B, an Ultrasound Doppler probe connected to a ultrasound machine; C, a three-lead ECG; D, a Laser Doppler flux probe; E, a Finometer probe; F, an ultrasound machine; G, an ice water bucket. Illustration: Øystein H. Horgmo, University of Oslo.

Brachial blood flow velocity and diameter were measured using a 9MHz linear transducer connected to a Vivid E9 ultrasound machine (Vingmed GE, Horten, Norway). A custom-made mechanical arm attached to the table ensured the transducer remained in a fixed position over the right brachial artery throughout the experiments (Fig 1). The ultrasound transducer (probe) position was fine‐tuned during the measurements with a micrometer screw connected to the mechanical arm, and the probe was held in the same position for the duration of the test. A pneumatic vascular cuff (Hokanson SC5 13 x 53 cm, Hokanson, Bellevue, Washington, USA) was placed on the lower right arm distally to the ultrasound probe to induce reactive hyperemia, thereby creating a flow stimulus. All measurements were performed by the same experienced operator.

### Data analysis

Analog signals from the Finometer, laser Dopplers and ECG were sampled at 300 Hz using custom-made software (REGIST 3). The signals were fed into a personal computer for further beat-by-beat analysis.

Images and spectral Doppler signals from the Vivid E9 machine were analyzed using custom-designed edge-detection and wall-tracking software (Brachial Analyzer, Medical Imaging Applications LLC, Coralville, Iowa, USA) [21]. The Brachial Analyzer software automatically measures arterial diameter, blood flow velocity, and FMD. The software allows users to identify the region of interest where the artery lumen appears most clearly. The FMD was defined as the maximum percentage change in artery diameter from baseline to peak during the three minutes after cuff deflation [4].

### Statistical analysis

Statistical analyses have been performed in SigmaPlot 14.0 (Systat software Inc. San Jose, CA, USA). The normality test (Shapiro-Wilk) failed for some variables. We therefore use the median (25th- 75th percentile), rather than the mean, in our data. Non-parametric statistics (Kruskal-Wallis One Way Analysis of Variance on Ranks) were used to test for significant differences between the different protocols. A value of P < 0.05 was accepted as statistically significant. The primary outcome in the present study was that changes in FMD, and a reduction in FMD of 2% or more were considered clinically significant. To be able to detect differences in FMD at this level, a minimum of 16 subjects were needed, given a power of 80% and 5% significance level.

## Results

Table 1 shows the 17 participants’ anthropometric data. We observed no complications during the study. None of the participants reported any pain during occlusion of the lower arm, which was examined immediately after completion of each experiment. One participant’s results were excluded in the final analysis due to low quality ultrasound measurements.

**Table 1:** Demographic and anthropometric data, n = 17^†^.

Age (yrs)	23.5	(23.0–24.8)
Body mass (kg)	76.5	(63.8–84.5)
Stature (cm)	177	(169.5–185.8)
BMI (kg/m^2^)	23.1	(21.5–25.0)
Physical activity (hours/week)	2.5	(2.0–4.0)
Heart rate (beats/min)	54.5	(46.0–64.3)
Systolic blood pressure (mm Hg)	122.5	(113.8–128.5)
Diastolic blood pressure (mm Hg)	66.0	(60.0–71.5)
Mean arterial pressure (mm Hg)	84.0	(79.0–90.3)

Values are median (25^th^ and 75^th^ percentiles).

† = 10 males and 7 females and BMI = Body Mass Index

### Flow-mediated vasodilation response

**Fig 2:**
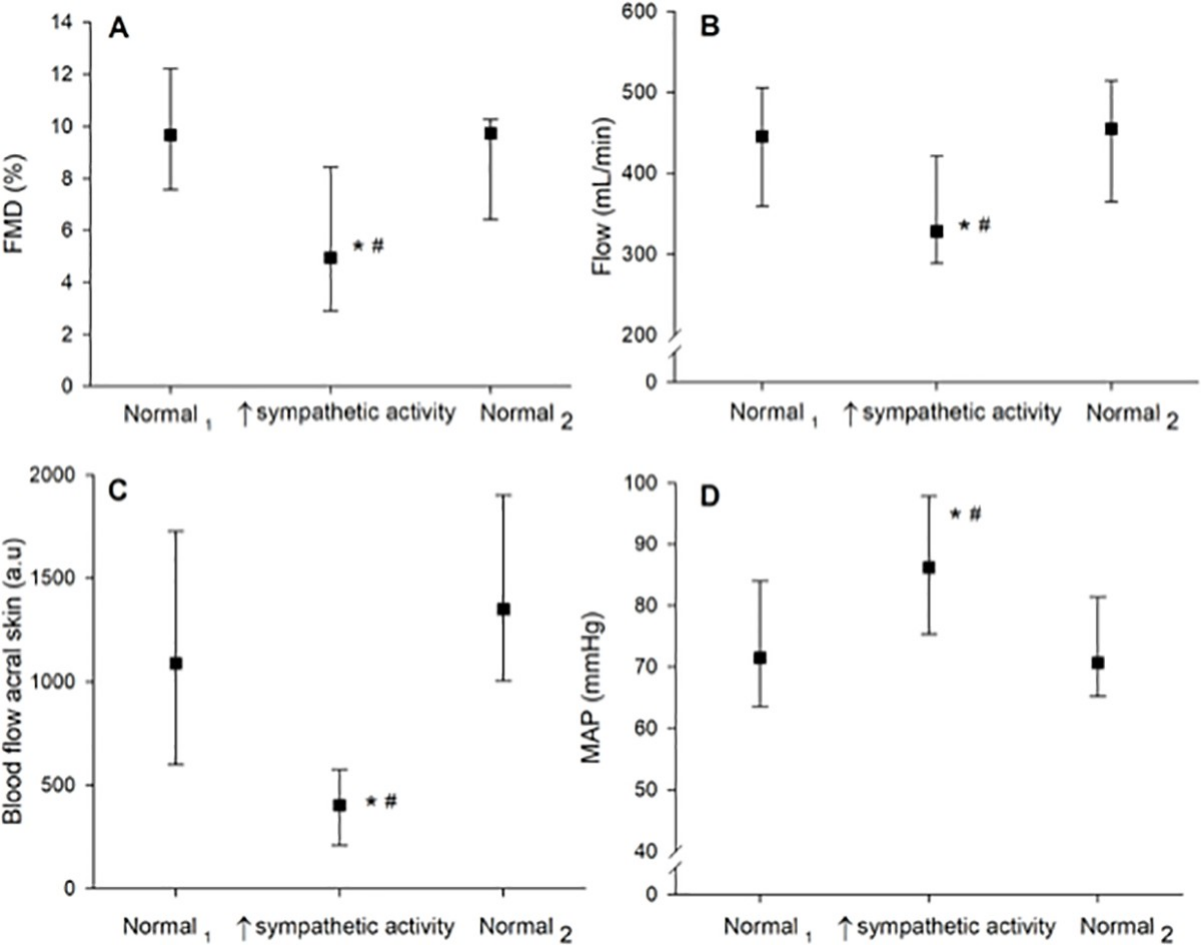
Physiological parameters. Changes in physiological parameters were measured during three sequences separated by 20-minute wash-out periods in the following order: during baseline (Normal_1_), during ice water exposure (sympathetic activity), and after ice water exposure (Normal_2_). Panel A: Change in %FMD; Panel B: Change in blood flow of the right brachial artery; Panel C: Change in pulp skin blood flow of the left index fingers; D: Change in mean arterial pressure (MAP). Values are median (25^th^ and 75^th^ percentiles). The marks * and # denote significance level (P < 0.01) from baseline (Normal_1_) and post-ice water exposure (Normal_2_), respectively.

FMD responses during normal non-stress conditions before and after exposure to ice water were (Normal_1_) 9.7 (7.6–12.2) % and (Normal_2_) 9.7 (6.4–10.3) %, respectively (P > 0.05). FMD decreased after ice water exposure to 4.9 (2.9–8.4) %, (P = 0.003) (Fig 2). The reduced FMD response was associated with reduced reactive hyperemia during ice water exposure.

### Change in blood flow of the peripheral vasculature

There was a significant reduction in brachial blood flow during ice water exposure (P = 0.005). The median of the mean blood flow following the first 60 s after cuff deflation was obtained during non-stress conditions: (Normal_1_) 446 (359–506) mL/min and (Normal_2_) 455 (365–515) mL/min (P > 0.05). The median of the mean brachial blood flow the first 60 s following ice water exposure was significantly lower than during the non-stress situations: [328 (289–421) mL/min] Normal_1_ and Normal_2_ (both P < 0.005 (Fig 2)).

### Hemodynamic response

The median of the mean arterial pressure (MAP) for the 16 participants during the first 60 s after cuff-deflation during non-stress conditions was 72 (64–84) mm Hg (Normal_1_) and 71 (65–81) mm Hg (Normal_2_). The median of the mean arterial pressure (MAP) the first 60 s during exposure to ice water was 86 (75–98) mm Hg (P = 0.011) (Fig 2).

We observed significant changes in heart rate (HR) between non-stress conditions and ice water during the first 60 s after cuff-deflation: 55 (49–60) bpm (Normal_1_) and 54 (47–60) bpm (Normal_2_). This compared to 67 (59–69) bpm during ice water exposure (P = 0.001).

Skin blood flow of the finger pulps the first 60 s after cuff-deflation was significantly lower during ice water exposure 404 (210–575) AU (P = 0.001), compared to non-stress conditions: 1089 (598–1730) AU (Normal_1_) and 1350 (1004–1904) AU (Normal_2_) (both P > 0.05 compared to baseline) (Fig 2).

We observed no significant correlation between the level of physical activity and sympathetic activity (r^2^ = 0.0014, P > 0.05)

## Discussion

The main finding in the present study was that, among healthy volunteers, increased sympathetic activity induced by foot ice water exposure resulted in reduction in the brachial artery FMD response compared to non-stress conditions. Moreover, the reduced FMD response was associated with lower peak reactive hyperemia during exposure to ice water.

Impaired brachial artery FMD has been reported in several recent studies as a parameter of endothelial dysfunction [22, 23]. The present study demonstrates that sympathetic activity influences the reactive hyperemia response and shear stress after occlusion, and significantly reduces the brachial artery FMD response. Our data is confirmed by Hijmering et al. [17], who have reported similar findings. Hijmering et al. [17] induced increased sympathetic tone by baroreceptor unloading, using a lower body negative pressure chamber. In that study, the researchers found that concomitant sympathetic stimulation significantly impaired the brachial artery FMD response in healthy subjects [17]. Further, the authors also found that infusion of phentolamine, a local regional alpha-adrenergic blockade, prevented the FMD response during sympathetic stimulation. The endothelium’s response to exogenous nitric oxide, by contrast, was unaffected during application of lower body chamber pressure [17]. In sum, the researchers found that increased sympathetic activity affected peripheral circulation independent of endothelial function.

The findings in the present study suggest, in accordance with Hijmering et al. [17] and Dyson et al. [15], that increased sympathetic outflow attenuates the brachial FMD response. Symptomatic treatment modalities that affect sympathetic outflow (e.g., beta-blockers and anesthesia tailored to inhibit sympathetic nervous system) may therefore also result in increased brachial artery FMD.

The present study’s findings indicate that the previously reported reduction in endothelial function after surgery [11] may be at least partly explained by increased post-operative sympathetic activation, leading to lower hyperemic flow and shear stress. Further research is needed to reveal the interaction between sympathetic activation and shear-mediated nitric oxide release by the endothelial cells.

Our findings are also in accordance with a recent clinical study by Dedichen et al. [12], finding reduced shear forces and brachial artery FMD response post-operatively. Additionally, the authors [12] observed reduced resting blood flow and hyperemic flow post-surgery without any change in baseline brachial artery diameter. In that study, the authors concluded that the reduction in brachial artery FMD after surgery, by previous authors taken to represent endothelial dysfunction, may be at least partly due to reduced hyperemic flow post-operatively [12]. Interestingly, endothelial function measured by brachial artery FMD follows diurnal variation, as it is lower in the morning than in the afternoon in healthy volunteers [24–27]; in patients with hypertension [28]; and in patients with variant angina [29]. In other words, FMD is low when sympathetic tone is known to be high [30]. Moreover, acute activation of the sympathetic nervous system has been demonstrated to attenuate the FMD response in several studies [16, 17].

The FMD response may depend on the external stimuli applied to induce sympathetic stress [15]. If increased sympathetic outflow leads to reduced blood flow and an impaired FMD response, FMD as a surrogate marker of endothelial function should be considered carefully in studies evaluating patients undergoing major surgery, when sympathoadrenal activity increases [31]. The results from the present study may therefore have consequences for patients undergoing brachial artery FMD assessments after major surgery or when the autonomic nervous system is altered [31, 32]. In sum, future studies should adjust for blood flow when assessing the brachial artery FMD response.

### Methodological considerations

In the present study, we investigated the effect of sympathetic stress on blood flow and FMD response in healthy volunteers. Whether our findings in healthy volunteers also apply to a patient population is a question for further investigations. Additionally, we did not measure plasma norepinephrine, epinephrine, or cortisol to quantify the participants’ sympathetic stress levels before and after the experiments with ice water. Nevertheless, cold-water submersion has been demonstrated to result in an up to four-fold elevated plasma noradrenaline concentration [15, 33]. Future studies should include these measurements to allow for comparison to blood values from patients undergoing major surgical interventions. Further, we did not measure cardiac output or venous pressure in the present study. We are therefore unable to elaborate the exact mechanisms of action for the reduction in blood flow and FMD response. It has been demonstrated that diminished FMD response is not a general response to increased sympathetic outflow [15]. It is possible that inducing increased sympathetic activity by other means, for example, by the hot water immersion test, would result in a different FMD response. Also, we did not examine endothelium-independent vasodilation using nitroglycerine for comparison to our FMD measurements.

High levels of physical activity increase vagal tone, which may theoretically blunt the sympathetic-induced responses. In the present study, we did not observe any correlation between level of physical activity and degree of sympathetic activation (i.e. an effect from vagal activation) (S1 Fig). Finally, this study was performed on healthy young volunteers and not a patient population, which may yield a different result.

## Conclusions

In this study, we show that increased sympathetic activity induced by ice water decreased resting blood flow, reactive hyperemia, and brachial artery FMD response in healthy volunteers. Our observations suggest that sympathetic activity may be a confounding factor in FMD studies demonstrating reduced endothelial function postoperatively. The observed decrease in FMD may result—at least partially—from reduced arterial shear stress caused by a sympathetic activity-induced reduction in blood flow. Our observations indicate that increased sympathetic tone, such as after major surgery, may attenuate the FMD response. Future clinical studies investigating brachial artery FMD as an indicator of endothelial dysfunction should adjust for blood flow when interpreting the FMD response.

## Supporting information

S1 Fig The relationship between physical activity and sympathetic activity response during the cold pressor test. The figure shows the correlation between physical activity and the degree of sympathetic activation (and by extension, the effect of vagal activation) among all healthy volunteers (n = 16). The figure shows the sympathetic response on the left hand (non-occluded hand) during foot ice water submersion, assessed as changes in pulp skin blood flow (Laser Doppler flux). The Y-axis illustrates the percentage of normal pulp skin blood flow during ice water exposure. The Y-axis' relative changes in pulp skin blood flow are calculated as 60 s of skin blood flow after submersion in ice water, divided by 60 s of skin blood flow pre-submersion. Pulp skin blood flow measurements are normalized (100%) to the average values pre-submersion. A high level of sympathetic acitivity corresponds to a low percentage. The X-axis shows the participants’ physical activity level, expressed as training hours/week (0–10 h).
(PDF)

S1 File The raw dataset for all participants. (XLSX)
