# Correction: The effects of sympathetic activity induced by ice water on blood flow and brachial artery flow-mediated dilatation response in healthy volunteers

**DOI:** 10.1371/journal.pone.0223798

**Published:** 2019-10-07

**Authors:** Kristian Magnus Gundersen, Christoffer Nyborg, Øyvind Heiberg Sundby, Jonny Hisdal

An incorrect version of Fig 1 was published in error, resulting in an illegible figure. The authors have provided an updated figure file. Please see an updated Fig 1 here.

**Fig 1:**
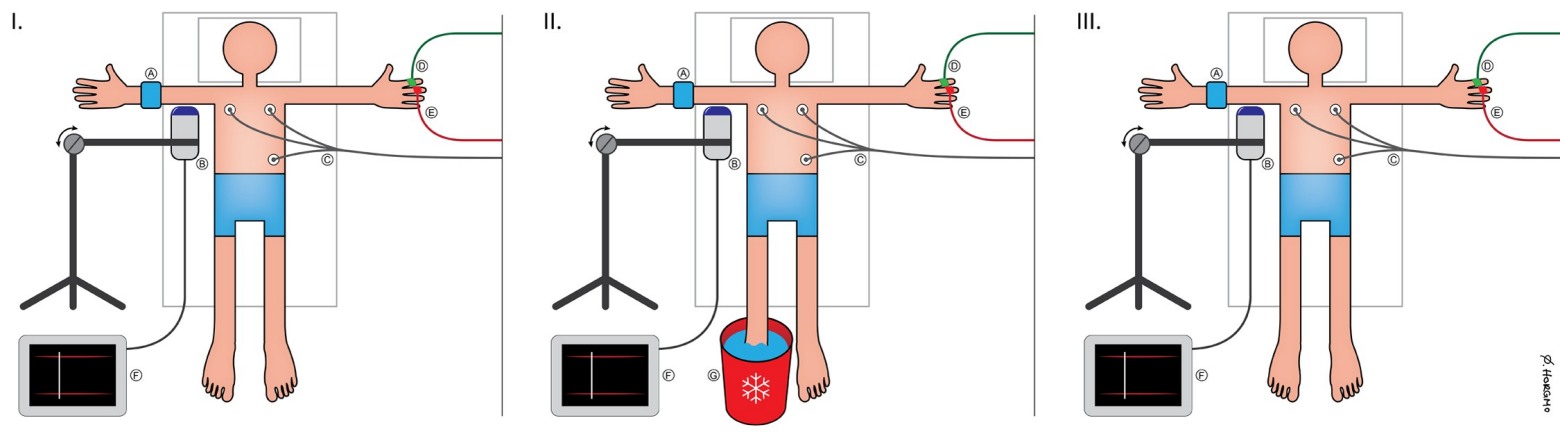
The experimental setup. I: Illustration of the experimental setup with probes attached to the upper extremites. I-II-III: A, a distal occlusion cuff attached to the lower right arm; B, an Ultrasound Doppler probe connected to a ultrasound machine; C, a three-lead ECG; D, a Laser Doppler flux probe; E, a Finometer probe; F, an ultrasound machine; G, an ice water bucket. Illustration: Øystein H. Horgmo, University of Oslo.
