# Supplementary figures and images for: The effects of sympathetic activity induced by ice water on blood flow and brachial artery flow-mediated dilatation response in healthy volunteers
Source: PLoS One. 2019 Sep 13;14(9):e0219814. doi: 10.1371/journal.pone.0219814 (PMC6743752; doi:10.1371/journal.pone.0219814)

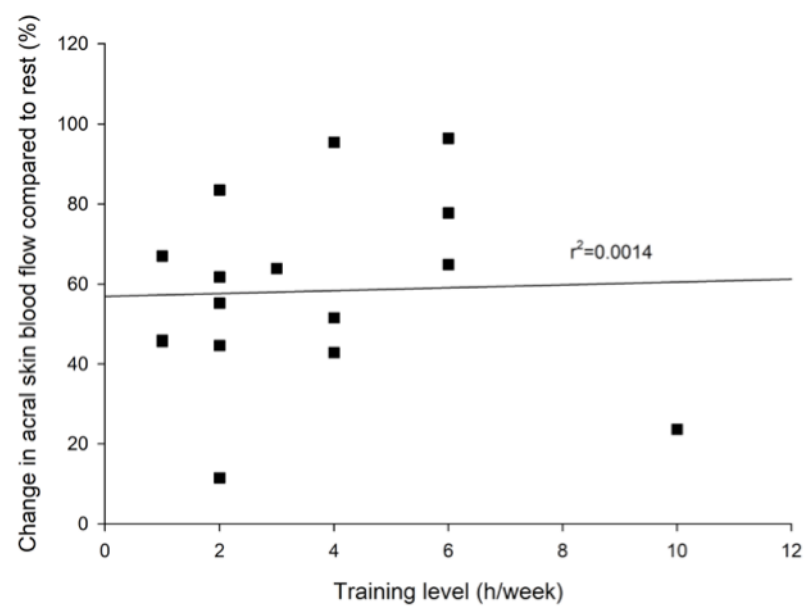

Supplement: S1 Fig — The figure shows the correlation between physical activity and the degree of sympathetic activation (and by extension, the effect of vagal activation) among all healthy volunteers (n = 16). The figure shows the sympathetic response on the left hand (non-occluded hand) during foot ice water submersion, assessed as changes in pulp skin blood flow (Laser Doppler flux). The Y-axis illustrates the percentage of normal pulp skin blood flow during ice water exposure. The Y-axis' relative changes in pulp skin blood flow are calculated as 60 s of skin blood flow after submersion in ice water, divided by 60 s of skin blood flow pre-submersion. Pulp skin blood flow measurements are normalized (100%) to the average values pre-submersion. A high level of sympathetic acitivity corresponds to a low percentage. The X-axis shows the participants’ physical activity level, expressed as training hours/week (0–10 h). (PDF) [file pone.0219814.s001.pdf]
